# A gender perspective on factors that influence outdoor recreational physical activity among the elderly

**DOI:** 10.1186/1471-2318-10-34

**Published:** 2010-06-08

**Authors:** Katarina Sjögren, Louise Stjernberg

**Affiliations:** 1 Faculty of Medicine, Department of Clinical Sciences in Malmö, General Practice Lund University, Sweden; 2 School of Health Science, Blekinge Institute of Technology, Karlskrona Sweden

## Abstract

**Background:**

Physical activity (PA) is part of a healthy lifestyle and prevents many chronic health problems, in addition to promoting mental health. PA performed outdoors has been found particularly good for promoting one's well-being. The aim of this study was to investigate the extent to which outdoor recreational PA was carried out during 1 year, and the factors influencing such activities from a gender perspective among persons ≥ 60 years of age.

**Methods:**

This study included 999 individuals 60-96 years of age living in the south eastern part of Sweden. Data collection was carried out during the years of 2001-2003. We measured the amount of regular light and/or intense outdoor recreational PA performed during the last year and determined the probability of performing PA as a function of 10 variables covering individual and socioeconomic factors.

**Results:**

Our results suggest that being independent physically and healthy enough to manage one's personal hygiene and having access to areas for country walks were the most important factors associated with the probability of engaging in outdoor recreational PA for both men and women. Despite the level of performance being almost equal for the sexes as two-thirds of both had performed outdoor recreational PA during the preceding year more factors, i.e., living alone, being unable to cover an unexpected cost, fear of being violated, and fear of falling, were associated with the possibilities of engaging in outdoor recreational PA among women. Also increasing age seems to affect activities among women negatively to a higher extent than men.

**Conclusion:**

Men and women seem to have different opportunities and needs with respect to performing PA. These considerations do not seem to be sufficiently taken into account today and improvements could be made concerning e.g., health-promoting activities suggested to the elderly by healthcare personnel and spatial planning within society. Promoting outdoor recreational PA that has restorative effects on well-being needs to focus on activities which are attractive and affordable for the majority of both men and women.

## Background

Being active throughout the majority of one's lifetime has an important influence on overall health and well-being. This paper has used the widely known definition of physical activity (PA) as "any bodily movement produced by the contraction of skeletal muscle that increases energy expenditure above a basal level" [1]. PA has been found to prevent many chronic health problems as well as to promote mental health and well-being [1-4]. Lower mortality rates occur among those who become physically active late in life as compared to those who were active in early life and stopped exercising. Although an increased vulnerability to chronic disease and disability is inherent in the aging process, evidence suggests that being physically active can influence the course of many frequently occurring diseases among the elderly [5].

Outdoor recreational PA, defined here as "to be outside in natural or cultural landscapes for well-being and encounters with nature without demands for competition" [6] has been shown to be particularly good for promoting well-being. PA performed outdoors in natural settings has been found to have restorative effects on people's health and to reduce mental fatigue [7,8].

Studies [9,10] have shown that there are factors that influence whether or not elderly people engage in PA, e.g., experience of pain or fatigue, lack of interest, or daily access to a car. To have a social network, including living with a partner, has been found to affect the performance of PA positively, while fear of falling and fear of violence have been found to have the opposite effect [11-14]. One marker of health and independence which is thought to affect the performance of PA is whether the person is able to bathe or shower independently [15,16]. Sweden has the oldest population in Europe; 5% of Sweden's population is > 80 years of age and the majority (64%) are women [17]. Women have consistently been found to have lower rates of participation in PA than men throughout life [4,18]. However, Bernstein et al. [19] suggested that women do not perform less PA than men, but are active in a different way through other kinds of activities, e.g., housework and walking. There may be a significant gender bias in the way PA is measured.

PA is a wide-ranging term that includes a variety of activities that may occur in many different settings, modes, and at varying frequencies and intensities. This complexity makes it difficult to measure. Over the last few decades, PA and related terms, e.g., exercise, have been defined somewhat inconsistently. The most valid assessment of an individual's level of activity remains a controversial and difficult area [20,21]. Furthermore, little is known about factors that influence outdoor recreational PA among the elderly, especially from a gender perspective.

Investments in health-promoting activities, such as outdoor recreational PA, are important and powerful factors in the continued healthful lives of the growing population of the elderly and it is therefore increasingly important to achieve a better understanding of PA behaviours among the elderly [22]. A better understanding about the factors that influence outdoor recreational PA among the elderly and differences between men and women would make it possible to plan appropriate health interventions that would hopefully lead to an active and healthy elderly population. The aim of this study was to investigate the extent to which outdoor recreational PA was carried out during 1 year, and the factors influencing such activities from a gender perspective among persons ≥ 60 years of age.

## Methods

### Study area, participants, and design

This study was part of the Swedish National Study on Aging and Care (SNAC), an interdisciplinary, longitudinal study that started in 2001. A more detailed description of the complete design and structure of the SNAC study are outlined by Lagergren et al. [23]. The characteristics of the sample that comprise the total SNAC study closely reflects those of the general population of elderly people in Sweden. Thus, the total SNAC sample may be perceived as representative of the elderly in Sweden [23]. This study includes baseline data collected between April 2001 and May 2003 from Karlskrona municipality, defined as a suburban region, with 60,600 inhabitants. Of these inhabitants, 19% are ≥ 65 years of age compared to 17.5% in the other areas of Sweden. Thirteen percent of inhabitants belong to the 65-79-year age group and 6% are ≥ 80 years of age, compared to 12.2% and 5%, respectively, in the other areas of Sweden [17]. Karlskrona is situated in the south eastern part of Sweden, is quite close the Baltic Sea, and has easy access to the countryside with both woodland and archipelago settings.

This study included individuals 60-96 years of age. The purpose of the data collection design was to receive a randomly selected sample, representing the old population in a broad variation of ages. Thus, those invited to participate were randomly selected members of the 60-, 66, 72-, and 78-year age cohorts, however, since the number of elderly decrease with increasing age, all surviving members of the 81-, 84-, 87-, 90-, 93-, and 96-year age cohorts were invited. The purpose of the data collection design was to receive a randomly selected sample, representing the old population in a broad variation of ages. For data management and analysis, the participants were divided into the following 4 age groups: 60-66, 72-78, 81-87, and 90-96 years. An invitation to participate in the study was sent by post. After 2 weeks, those who had not replied to the first invitation were contacted by telephone (three attempts). Those who agreed to participate were invited to the research centre; if they were unable to attend, a researcher visited them at their home. The participants completed a questionnaire that included items concerning sex, age, ability to bathe or shower independently, smoking habits, living status, level of education, being able to cover an unexpected cost, being part of a social network, whether the participant had abandoned outdoor recreational PA due to fear of violence or due to fear of falling, and access to areas offering country walks.

Outdoor recreational PA was measured by the following two survey questions: A) "How often have you performed PA with light intensity (e.g., walking in roads, in parks, or in the woods, short bicycle tours, light gymnastics, and golf or similar activities) during the last 12 months," and B) "How often have you performed PA more intensely (e.g., jogging, long and high intensity walking, heavy gardening, long bicycle tours, intense gymnastics, long-distance skating, skiing, swimming, ball games (not golf), or similar activities) during the last 12 months."

### Statistical analysis

Descriptive statistics were used to evaluate sample characteristics. The Pearson chi-square test was used to evaluate gender differences in variables covered in the questionnaire and to evaluate differences in characteristics between groups that performed outdoor recreational PA (light and/or more intense) in relation to all the other variables covered in the questionnaire.

Stepwise multiple binary logistic regression was used to evaluate the associations between various independent variables and the performance of outdoor recreational PA. The dependent variable was measured by combining answers to the two questions about outdoor recreational PA (light and/or more intense activities during the last 12 months) on a binary scale, as follows 0 = never to 1-3 times/month, and 1 = several times per week to every day. Thus, the relative input of light and intense activities was the same. Simultaneous relationships between the dependent variable and all independent variables were modelled and evaluated using odds ratio (OR). Two different models were tested separately for both men and women. In the first model, all independent variables from the questionnaire were included (Table 1). The next model included variables for which a significant impact on the probability of performing outdoor recreational PA existed between the independent variables that included more than 5 participants in each combined group, and also evaluated association for interaction between these variables, with a *p*-value < 0.005. The statistical significance of the variables was determined using a 95% confidence interval (CI). Data were computerised and analysed using SPSS 17.0 for Windows.

**Table 1:** Independent variables included in the first model in the logistic regression analysis evaluating factors with a significant influence on performance of outdoor recreational physical activity among elderly men and women.

Variables	Coded as			
	0		1	
Able to bathe or shower independently	able^1^		not able	
Living status	cohabiting^2^		single^3^	
Level of education	lower^4^		higher^5^	
Personal financial situation	able^6^		not able^7^	
Being part of a social network	yes		no	
Abandoned outdoor recreational PA* during the evenings due to fear of violence	yes^8^		no^9^	
Abandon outdoor recreational PA* due to fear of falling	yes^8^		no^9^	
Have access to areas for country walks	access		no access	
Smoking habits	smoker^10^		non-smoker^11^	

	**Dummy variables**			
Age stratified into age groups	60-66	72-78	81-87	90-96

^1 ^totally on my own/need help to wash my back

^2 ^married/living with a partner

^3 ^widow/widower/unmarried/divorced

^4 ^uncompleted elementary school, nine-year compulsory school, vocational training

^5 ^senior high school, higher certificate, university degree, postgraduate study

^6 ^able to cover an unexpected cost within a week

^7 ^not able to cover an unexpected cost within a week

^8 ^often/rather often

^9 ^never/seldom

^10^smokes regularly/sometimes

^11 ^stopped smoking/never smoked

* Physical activity

### Ethics

The study was approved by the Research Ethics Committee at Lund University. Informed written consent was obtained from all participants.

## Results

### Participant characteristics

In total, 2312 people were invited to participate and 1402 (61%) agreed to do so. Of the participants were 585 men and 817 women. Of the 910 non-participants, 355 (39%) were men and 555 (61%) were women. The reasons given for not participating were unwillingness 756, (83%), or considering themselves too sick to participate, 92 (10%). Contact could not be made with 62 people (7%). Of the 1402 persons who agreed to participate, 999 (71%) answered all of the questions in the questionnaire, and thus constitute the sample included in this study. Typically, those participants not answering all of the questions were ≥ 81 years of age and suffered from illness or functional impairment. Participant characteristics and gender differences are presented in Table 2. The number of participants according to age and gender and participation in outdoor recreational PA is presented in Table 3.

**Table 2:** Participant characteristics and gender differences among elderly people included in a study of outdoor recreational physical activity.

	Men n = 451 (45%)	Women n = 548 (55%)	Total n = 999	Pearson chi-square
**Age (years)**				
Mean	74	75	74	
Median	72	77	74	
Range	60-96	60-96	60-96	
				
**Able to bathe or shower independently**				
Yes	435 (96)	504 (92)	939 (94)	
No	16 (4)	44 (8)	60 (6)	*p *= 0.003
				
**Smoking**				
Yes	60 (13)	55 (10)	115 (12)	
No	391 (87)	493 (90)	884 (88)	n.s.
				
**Cohabiting**				
Yes	327 (73)	239 (44)	566 (57)	
No	124 (27)	309 (56)	433 (43)	*p *= 0.000
				
**Level of education**				
Lower education	351 (78)	471 (86)	921 (92)	
Higher education	100 (22)	77 (14)	78 (8)	*p *= 0.011
				
**Being able to cover an unexpected cost**				
Yes	396 (88)	430 (78)	826 (83)	
No	55 (12)	118 (22)	173 (17)	*p *= 0.000
**Being part of a social network**				
Yes	236 (52)	312 (57)	544 (55)	
No	215 (48)	236 (43)	447 (45)	n.s.
				
**Abandoning outdoor recreational physical activity during the evenings due to fear of violence**				
Often	11 (2)	76 (14)	87 (9)	
Never	440 (98)	472 (86)	912 (91)	*p *= 0.000
				
**Abandoning outdoor recreational physical activity due to fear of falling**				
Yes	15 (3)	59 (11)	74 (7)	
No	436 (97)	489 (89)	925 (93)	*p *= 0.000
				
**Access to areas for country walks**				
Yes	401 (89)	449 (82)	85 (15)	
No	50 (11)	99 (18)	850 (85)	*p *= 0.002

**Table 3:** Participation in outdoor recreational physical activity according age and gender.

	Never/1-3 times per month		Several times per week/every day		Total
	n (%)*	(%)**	n (%)*	(%)**	n (%)*
**Men (n = 451)**					
60-66 years (n = 167)	55 (12)	(33)	112 (25)	(67)	167 (37)
72-78 years (n = 120)	32 (7)	(27)	88 (20)	(73)	120 (27)
81-87 years (n = 145)	55 (12)	(38)	90 (20)	(62)	145 (32)
90-96 years (n = 19)	13 (3)	(68)	6 (1)	(32)	19 (4) 19 (4)
Total	155 (34)		296 (66)		451 (100)
			
**Women (n = 548)**					
60-66 years (n = 183)	29 (5)	(16)	154 (28)	(84)	183 (33)
72-78 years (n = 149)	46 (8)	(31)	103 (19)	(69)	149 (27)
81-87 years (n = 184)	89 (17)	(48)	95 (17)	(52)	184 (34)
90-96 years (n = 32)	21 (4)	(66)	11(2)	(34)	32 (6)
Total	185 (34)		363 (66)		548 (100)
			
**All (n = 999)**					
60-66 years (n = 350)	84 (8)	(24)	266 (27)	(76)	350 (35)
72-78 years (n = 269)	78 (8)	(29)	191 (19)	(71)	269 (27)
81-87 years (n = 329)	144 (14)	(44)	185 (19)	(56)	329 (33)
90-96 years (n = 51)	34 (3)	(67)	17 (2)	(33)	51 (5)
Total	340 (34)		659 (66)		999 (100)

* Percentage referring to all participants

** Percentage within the age group

### Differences between groups and factors that were significantly associated with performance of outdoor recreational PA among men

Factors that were significantly associated with outdoor recreational PA among men were being able to bathe or shower independently, being a non-smoker, and having access to areas for country walks (Table 4). The performance of outdoor recreational PA decreased significantly in the 72-78-year age group (Pearson chi-square, *p *= 0.038) and in the 90-96-year age group (Pearson chi-square, *p *= 0.001).

**Table 4:** Differences between men and women and factors that were significantly associated with the performance of outdoor recreational physical activity (four-fold table).

Factor	Men Pearson chi-square *p*	Women Pearson chi-square *p*
Being able to bathe or shower independently	0.000	0.000
Being a non-smoker	0.031	0.170 n.s.
Having access to areas for country walks	0.002	0.000
Cohabiting or not	0.232 n.s.	0.000
Level of education	0.880 n.s.	0.000
Being able or unable to cover an unexpected cost	0.214 n.s.	0.014
Being part of a social network or not	0.576 n.s.	0.076 n.s.
Having fear of violence or not	0.888 n.s.	0.015
Having fear of falling or not	0.116 n.s.	0.000

### Differences between groups and factors that were significantly associated with performance of outdoor recreational PA among women

Factors that were significantly associated with outdoor recreational PA among women were being able to bathe or shower independently, cohabiting, having a higher level of education, being able to cover an unexpected cost, having no fear of violence, having no fear of falling, and having access to areas for country walks (Table 4). The performance of outdoor recreational PA decreased significantly in the 81-87-year age group (Pearson chi-square, *p *= 0.000) and in the 90-96-year age group (Pearson chi-square, *p *= 0.000) as compared to the 60-66-year age group.

### Logistic regression analysis

The final model for men involved 5 variables, including one interaction variable:

• Men 90-96 years of age as compared to men 60-66 years of age

• Being a non-smoker

• Able to bathe or shower independently

• Access to areas for country walks

• Men 90-96 years of age as compared to men 60-66 years of age and being a smoker

The final model for women involved 7 variables, including one interaction variable:

• Women 72-78 years of age as compared to women 60-66 years of age

• Women 81-87 years of age as compared to women 60-66 years of age

• Women 90-96 years of age as compared to women 60-66 years of age

• Able to bathe or shower independently

• Access to areas for country walks

• Level of education

• Women 81-87 years of age as compared to women 60-66 years of age and having access to areas for country walks

### Predictors of the probability for performing outdoor recreational PA among men and women

The variables that were significantly associated with an increased probability of performing outdoor recreational PA among men and women are presented in Table 5. Except for those 90-96 years of age, all other variables described increased the probability for performing outdoor recreational PA among men. For women, the probability decreased in all of the higher age groups. However, the interaction variable showed that if the participant was 81-87 years of age and had access to areas for country walks, the probability increased.

**Table 5:** Binary logistic regression predicting independent factors significantly associated with the probability of performing outdoor recreational physical activity among elderly men and women.

	Factor	Odds Ratio*	Confidence interval	*p*-value
**Men**				
	90-96 years^1^	0.291	0.101-0.841	0.023
	Being a non-smoker	1.941	1.105-3.409	0.021
	Being able to bathe or shower independently	11.057	2.398-50.991	0.002
	Having access to areas for country walks	2.220	1.191-4.138	0.012
				
**Women**	72-78 years^1^	0.469	0.274-0.804	0.006
	81-87 years^1^	0.411	0.235-0.717	0.002
	90-96 years^1^	0.200	0.081-0.498	0.001
	Being able to bathe or shower independently	6.820	2.831-16.432	0.000
	Having a higher level of education	2.293	1.121-4.690	0.023
	81-87 years and having access to areas for country walks	4.388	2.145-8.976	0.000

^1^Versus the 60-66-year-old age group

^2 ^Interaction variable measuring age group 81-87 years as compared to age group 60-66 years and having access to areas for country walks

*Covariates were adjusted to each odds ratio

## Discussion

The performance was almost equal between the sexes since two-thirds of both men and women had performed outdoor recreational PA during the preceding year. The main findings from this study indicate that being physically healthy enough to manage one's personal hygiene and having access to areas for country walks are the most important factors associated with the probability of performing outdoor recreational PA for both men and women. Even if no interaction was seen between age and gender the probability for activities decreased with advancing age in all age groups among women. Increasing seems to affect women more than men. Smoking habits among men and educational level among women were also important factors affecting the probability of engaging in PA. Living alone, being unable to cover an unexpected cost, fear of being violated, and fear of falling were factors that negatively affected the performance of outdoor recreational PA among women. Thus, there were more factors associated with limitations for women to perform outdoor recreational PA than men.

In contrast to other studies [24,25], we found women were just as physically active as men. It would be remarkable if women were less active and differed in behaviour from men with respect to PA, when generally they exhibit more positive health behaviours than men. Such statements might be explained by the fact that many studies have focused on activities strongly associated with sports and exercise, traditionally performed mostly by men, and less on activities performed more by women, such as housework, gardening, walking, and biking, [26]. We included a wide spectrum of activities performed outdoors, offering a broader opportunity to include more physical activities and this might explain our findings, i.e., that women are almost equally physically active as men.

Not surprisingly, the marker we used for health and independence, i.e., being able to bathe or shower independently, was found, to positively affect the performance of outdoor recreational PA among both men and women. These findings are in agreement with other studies [13] which showed that somatic health status is clearly associated with PA, and [27] that participation in PA is influenced only by physical limitations among the elderly.

Our findings, stressing the importance of having access to areas for country walks agree with data from previous studies [28] showing that the performance of PA is significantly influenced by access to a park or a recreation centre. Men were found to have greater access to such areas than women, which might have a financial explanation. Men have a more favourable financial situation e.g., owning a car and having a driving license are more common among elderly men than women, and this could make it easier for men to engage in outdoor recreational PA [10]. Men also have higher average pensions than women and are better able to cover unexpected costs [29]. These economic differences are expected to persist since women remain more likely to work part-time and more likely to be lower-wage earners, both of which have a negative effect on their pensions. Women therefore might need supplementary support from society to access recreational areas, especially as we found that women in the 81-87 age group have more outdoor recreational PA if they have access to areas for country walks.

Our results, show that smoking habits among men and educational level among women are important factors which affect the probability of performing outdoor recreational PA. Studies [14,30] have shown a negative correlation between smoking and PA that is confirmed by our results, but only among men. However, in Sweden smoking is more common among women than among men, a fact that has to be taken into account when planning health-promoting activities. The number of smoking participants in this study was low, which reflects the smoking habits among Swedish adults. In fact, Sweden has Europe's smallest proportion of daily smokers among men. In 2003, 17% of men and 18% of women aged 16-84 years were daily smokers [31]. Individuals with less secondary education tend not to be as physically active as more educated individuals, and formerly women tended to be less educated than men [24,25]. In this study, women with a higher level of education were more likely to perform outdoor recreational PA than women with a lower level of education.

Other factors that affected the performance of women to a higher extent than that of men, were cohabiting and fear of violence or falling. These findings are in agreement with the findings of Lee [32], who showed that women are more likely to be in situations less conducive to PA than men both with respect to living conditions and the financial situation. However, unmarried men have been found to be more physically inactive than unmarried women [14]. As others suggest, fear of violence seems to have a negative effect on the performance of PA among elderly women [13]. Fear of falling is common among the elderly and is one psychological barrier to performing PA. Both of these factors imply a risk factor of developing the other [11]. Fall-induced injuries among the elderly are an increasing public health concern in modern societies with aging populations [33] and PA is an important factor in preventing such injuries. The importance of PA in this respect needs to be stressed. Accordingly, it is important to create supportive outdoor spaces, such as spaces that are easy for the elderly to use, contribute to more active lifestyles which facilitate life satisfaction and health [34], promote PA, and prevent injuries.

Compared with the 60-66-year age group, outdoor recreational PA decreased significantly in all older age groups among women. No significant decrease was seen among men until the participants reached the 90-96-year age group. Suggesting different kinds of activities might provide the elderly with more opportunities to remain physically active and/or influence PA behaviour.

The number of the oldest old will continue to increase due to the size of the aging population and increasing longevity. Data from this study help to emphasise the importance of implementing preventive measures in order to add health to longer lives. In contrast to other studies [35] which have excluded participants > 70 years of age, assuming that the PA patterns depended largely on their health status, we included those who were 90-96 years of age, even if they only comprised a small fraction of the participants.

PA does not have to be vigorous to yield health benefits. Every day activities, such as walking and gardening, have positive health effects [4]. Our purpose was to investigate whether there was any outdoor recreational PA, and thus the answers to the two survey questions on light and more intense outdoor recreational PA during the last 12 months were put together and dichotomized. Other studies [3,25] measured moderate PA, which was not specifically evaluated in our study and might have provided more information. Aware that many elderly people perform PA indoors, we chose specifically to study PA performed outdoors because of the health benefits known to result from interacting with nature [7,8,36].

Our definition of PA included a broad range of both organised and unorganised activities. Participants were given examples of activities on the questionnaire and were allowed to evaluate the various activities that they participated in over the course of 1 year. The intensities and types of activities can vary widely during a year and the seasons are likely to influence both the activity level and the activities chosen. In the questionnaire, the participants only reported in general terms the extent to which they had performed PA during the year. The variation in the types of activities design could have lead to bias in our results. The survey question used to assess PA should be seen as a fairly simple measure of PA and may not be as strong as more advanced and validated instruments of PA for the elderly. The retrospective nature of the study may also entail bias. For some elderly people the memory of PA over a period of a whole year is difficult, and might not offer adequate insight. This study had a rather high share of non-participants (response rate, 43.2%). Non-participants increase threats to the external validity, and generalizing the findings has to be done with caution [37]. Owing to the formulations of the questions on PA, people with major functional limitations did not find them relevant to answer. Therefore, the response rate among those > 81 years was low, that has also affected the total response rate. We included participants 90 years of age and older. Even though this group comprised a small fraction of the participants (51/999; 5%), they are an important group since the numbers of the oldest old will continue to increase due to the size of the aging population and increasing longevity. The strengths of this study are that it was based on a random age-stratified sample of elderly people, the number of participants was high, the age of the participants ranged from 60-96 years, and the distribution according to gender was almost equal (54.9% women and 45.1% men).

## Conclusion

Being independent and healthy enough to manage one's personal hygiene and having access to areas for country walks emerged as the most important factors associated with the probability of performing outdoor recreational PA for both men and women. Even if the performance was almost equal between the sexes since two-thirds of both men and women had performed outdoor recreational PA during the preceding year, we found that there were more factors associated with possibilities of performing outdoor recreational PA among women. Also increasing age seems to affect activities among women negatively to a higher extent than men. Men and women seem to have different opportunities and needs to perform PA. Our results lead us to conclude that these considerations might not be sufficiently taken into account today and could be improved, i.e., in the health-promoting activities suggested to elderly people by healthcare personnel, as well as in spatial planning within society. More studies about gender differences are needed in this field. The promotion of outdoor recreational PA, which also has restorative effects on well-being, needs to focus on activities which are attractive and affordable for the majority of both men and women.

## Competing interests

The authors declare that they have no competing interests.

## Authors' contributions

KS and LS analyzed the data, drafted the paper and both authors contributed to the final manuscript.

## Pre-publication history

The pre-publication history for this paper can be accessed here:

http://www.biomedcentral.com/1471-2318/10/34/prepub

## References

[B1] Physical Activity Guidelines Advisory Committee Physical Activity Guidelines Advisory Committee Report, 2008 2008 Washington DC: Department of Health and Human Services

[B2] Bhargava A A longitudinal analysis of the risk factors for diabetes and coronary heart disease in the Framingham Offspring Study Population Health Metrics 2003 1 3 10.1186/1478-7954-1-3 12773213 PMC156626

[B3] Keim NL Blanton CB Kretsch MJ America's Obesity Epidemic: Measuring physical activity to promote an active lifestyle Journal of the American Dietetic Association 2004 104 1398 1409 10.1016/j.jada.2004.06.005 15354157

[B4] US Department of Health and Human Services The Surgeon General's report on physical activity and health 1996 Washington DC: US Government Printing Office

[B5] Paffenberg RS Hyde RT Wing AL Hsieh CC Physical activity, all-cause mortality and longevity of college alumni New England Journal of Medicine 1986 314 605 613 3945246 10.1056/NEJM198603063141003

[B6] Fredman P Sandell K Boman M Emmelin L Lundmark L Romlid U Outdoor recreation in change - Landscapes, experiences, planning and development. Program plan 2006-08-18 2006 http://www.friluftsforskning.se/download/18.6983d52510f51a3d56e80002728/Programplan_Friluftsliv+i+f%C3%B6r%C3%A4ndring_060818.pdf Retrieved October 5, 2009

[B7] Frumkin H Beyond toxicity human health and the natural environment American Journal of Preventive Medicine 2001 20 234 240 10.1016/S0749-3797(00)00317-2 11275453

[B8] Kaplan R Kaplan S The experience of nature: A psychological perspective 1989 Cambridge University Press

[B9] Cooper KM Bilbrew D Dubbert PM Kerr K Kirchner K Health barriers to walking for exercise in elderly primary care Geriatric Nursing 2001 22 258 262 10.1067/mgn.2001.119470 11606904

[B10] Crombie IK Irvine L Williams B McGinnis AR Slane PW Alder EM McMurdo MET Why older people do not participate in leisure time physical activity: a survey of activity levels, beliefs and deterrents Age and Ageing 2004 33 287 292 10.1093/ageing/afh089 15082435

[B11] Bruce DG Devine A Prince RL Recreational physical activity levels in healthy older women: The importance of fear of falling Journal of the American Geriatrics Society 2002 50 84 89 10.1046/j.1532-5415.2002.50012.x 12028251

[B12] Friedman SM Munoz B West SK Rubin GS Fried LP Falls and fear of falling: which comes first? A longitudinal prediction model suggests strategies for primary and secondary prevention Journal of the American Geriatrics Society 2002 50 1329 1335 10.1046/j.1532-5415.2002.50352.x 12164987

[B13] Piro NF Naess O Claussen B Physical activity among elderly people in a city population: the influence of neighbourhood level violence and self perceived safety Journal of Epidemiology and Community Health 2006 60 626 632 10.1136/jech.2005.042697 16790836 PMC2566241

[B14] Zimmerman E Ekholm O Grönbaek M Curtis T Predictors of changes in physical activity in a prospective cohort study of the Danish adult population Scandinavian Journal of Public Health 2008 36 235 241 10.1177/1403494808086982 18519291

[B15] Landi F Onder G Carpenter I Cesari M Soldato M Bernabei R Physical activity prevented functional decline among frail community-living elderly subjects in an international observational study Journal of Clinical Epidemiology 2007 60 518 524 10.1016/j.jclinepi.2006.09.010 17419963

[B16] Stessman J Hammerman-Rozenberg R Maaravi Y Azoulai D Cohen A Strategies to enhance longevity and independent function: the Jerusalem longitudinal study Mechanisms of Ageing and Development 2005 126 327 331 10.1016/j.mad.2004.08.024 15621214

[B17] Statistiska Centralbyrån [Statistics Sweden] http://www.karlskrona.se/Global/karlskrona%20kommun/Dokument/Om%20kommunen/befolkning/Karlskron_FAKTA2008_Eng.pdf?esplanguage=sv Retrieved October 5, 2009

[B18] Leslie E Fotheringham MJ Owen N Bauman A Age-related differences in physical activity levels of young adults Medicine & Science in Sports & Exercise 2001 33 255 258 10.1097/00005768-200102000-00014 11224815

[B19] Bernstein S Costanza MC Morabia A Physical activity of urban adults: a general population survey in Geneva Sozial-und Präventivmedizin 2001 46 49 59 10.1007/BF01318798 11320913

[B20] Riddoch C Ewles L Physical activity in key topics in public health - essential briefings on prevention and health promotion 2005 London: Churchill Livingstone

[B21] World Health Organization Obesity: Preventing and Managing the Global Epidemic. Report of a WHO Consultation on Obesity Geneva 2000 World Health Organization 11234459

[B22] Communication from the European Commission The demographic future of Europe - from challenge to opportunity 2006 Brussels, European Commission

[B23] Lagergren M Fratiglioni L Hallberg IR Berglund J Elmstahl S Hagberg B Holst G Rennemark M Sjölund BM Thorslund M Wiberg I Winblad B Wimo A A longitudinal study integrating population, care and social service data. The Swedish National Study on Aging and Care (SNAC) Aging Clinical and Experimental Research 2004 16 158 68 15195992 10.1007/BF03324546

[B24] Aslan D Özcebe H Temel F Takmaz S Topatan S Sahin A Ankan M Tannverdi B What influences physical activity among elders? A Turkish experience from Ankara, Turkey Archives of Gerontology and Geriatrics 2008 46 79 88 10.1016/j.archger.2007.03.001 17482688

[B25] Kaplan MS Newsom JT McFarland BH Lu L Demographic and psychosocial correlates of physical activity in late life American Journal of Preventive Medicine 2001 21 306 312 10.1016/S0749-3797(01)00364-6 11701302

[B26] Abel N Graf N Niemann S Gender bias in the assessment of physical activity in population studies Sozial-und Präventivmedizin 2001 46 268 272 10.1007/BF01593182 11582854

[B27] Paillard-Borg S Wang H-X Winblad B Fratiglioni L Pattern of participation in leisure activities among older people in relation to their health conditions and contextual factors: a survey in a Swedish urban area Ageing & Society 2009 29 803 821

[B28] Booth ML Owen N Bauman A Clavisi O Leslie E Social-cognitive and perceived environment influences associated with physical activity in older Australians Preventive Medicine 2000 31 15 22 10.1006/pmed.2000.0661 10896840

[B29] National Swedish Board of Health and Welfare Social rapport 2006. [Social report 2006] 2006 Stockholm: Norstedts Tryckeri

[B30] Varo JJ Martinez-Gonzalez MA De Irala-Estevez J Kearney J Gibney M Martinez JA Distribution and determinants of sedentary lifestyles in the European Union International Journal of Epidemiology 2003 32 138 146 10.1093/ije/dyg116 12690026

[B31] Boström G Chapter 9: habits of life and health Scandinavian Journal of Public Health 2006 34 199 228 10.1080/14034950600677287 16762911

[B32] Lee Y-S Gender differences in physical activity and walking among older adults Journal of Women and Aging 2005 17 55 70 10.1300/J074v17n01_05 15914419

[B33] Kannus P Niemi S Palvanen M Parkkari J Rising incidence of fall-induced injuries among elderly adults Journal of Public Health 2005 13 212 215 10.1007/s10389-005-0115-0

[B34] Sugiyama T Ward Thompson C Older people's health, outdoor activity and supportiveness of neighborhood environments Landscape and Urban Planning 2007 83 168 175 10.1016/j.landurbplan.2007.04.002

[B35] Van Lenthe FJ Brug J Mackenbach JP Neighbourhood inequalities in physical inactivity: the role of neighbourhood attractiveness, proximity to local facilities and safety in the Netherlands Social Science & Medicine 2005 60 763 775 10.1016/j.socscimed.2004.06.013 15571894

[B36] Irvine KN Warber SL Greening healthcare: practising as if the natural environment really mattered Alternative Therapies in Health and Medicine 2002 8 76 83 12233806

[B37] Kazdin AE Research design in clinical psychology 2003 fourth Boston: Allyn and Bacon

